# Simultaneous gastric outlet and small bowel obstruction due to phytobezoar: a rare case report

**DOI:** 10.1093/jscr/rjaf873

**Published:** 2025-11-05

**Authors:** Nuradin Mohamed Nur, Abdisalam Ismail Hassan, Abdinasir Artan Jubur, Mohamed Rage Ahmed, Shuayb Moallim Ali Jama

**Affiliations:** Faculty of Medicine and Health Sciences, Jamhuriya University of Science and Technology, Digfer Street, Hodan District, Mogadishu, Somalia; General Surgery Department, Mogadishu Somali-Türkiye Recep Tayyip Erdoğan Training and Research Hospital, Digfer Street, Hodan District, Mogadishu, Somalia; Faculty of Medicine and Health Sciences, Jamhuriya University of Science and Technology, Digfer Street, Hodan District, Mogadishu, Somalia; General Surgery Department, Mogadishu Somali-Türkiye Recep Tayyip Erdoğan Training and Research Hospital, Digfer Street, Hodan District, Mogadishu, Somalia; General Surgery Department, Mogadishu Somali-Türkiye Recep Tayyip Erdoğan Training and Research Hospital, Digfer Street, Hodan District, Mogadishu, Somalia; Radiology Department, Mogadishu Somali-Türkiye Recep Tayyip Erdoğan Training and Research Hospital, Digfer Street, Hodan District, Mogadishu, Somalia

**Keywords:** bezoars, phytobezoars, small bowel obstruction, gastrostomy, and enterotomy

## Abstract

Small bowel obstruction is a common surgical problem with a wide variety of causes, although bezoars are a rare etiology. Among them, phytobezoars, composed of undigested vegetable or fruit fibers, are the most frequent type. Their clinical presentation often mimics more common causes of intestinal obstruction, making accurate diagnosis challenging. We report the case of a 78-year-old woman who presented with an 8-day history of abdominal pain, nausea, and vomiting. Imaging revealed both gastric outlet and small bowel obstruction. At laparotomy, two phytobezoars were identified and successfully removed from the stomach and ileum. The patient recovered well and was discharged on postoperative day 6. This case highlights the importance of considering bezoars as a differential diagnosis in intestinal obstruction, as management may be surgical or conservative depending on the circumstances.

## Introduction

Small intestinal obstruction (SBO) is a frequent surgical condition that can have a variety of etiologies, including tumors, volvulus, intussusceptions, hernias, and postoperative adhesions (60%–80%). A proper differential diagnosis is crucial because the symptoms in all of these instances are similar and include abdominal pain, nausea, vomiting, constipation, and occasionally fever [1].

Less than 0.5% of people who undergo upper gastrointestinal endoscopy have bezoars in their stomachs, and 0.4% to 4.8% of all cases presenting with intestinal obstruction have bezoars in their small bowel [2, 3]. Phytobezoar, an accumulation of undigested fruit or vegetable fiber, discovered in the gastrointestinal tract [4]. In rare instances, they can form and hinder the stomach, resulting in ulceration, bleeding, or obstruction [5]. The most frequent type of bezoar is a phytobezoar, which usually obstructs the narrowest part of the small intestine [6].

The non-specific symptoms of SBO [1] complicate the diagnosis owing to the low incidence of bezoars. We report a rare case of combined gastric outlet and small bowel obstruction caused by phytobezoars managed surgically by laparotomy.

## Case presentation

A 78-year-old woman who had been experiencing abdominal pain, nausea, and vomiting for 8 days was brought into our emergency department. She described the pain as a continuously worsening ache for an hour, which had been mildly relieved by antiemetics in the earlier days.

Her vital signs on admission were stable; the patient appeared ill on physical examination, and abdominal examination revealed mild distension. The laboratory tests were within normal ranges on admission. The patient had no history of psychiatric or neurological disease.

**Figure 1:**
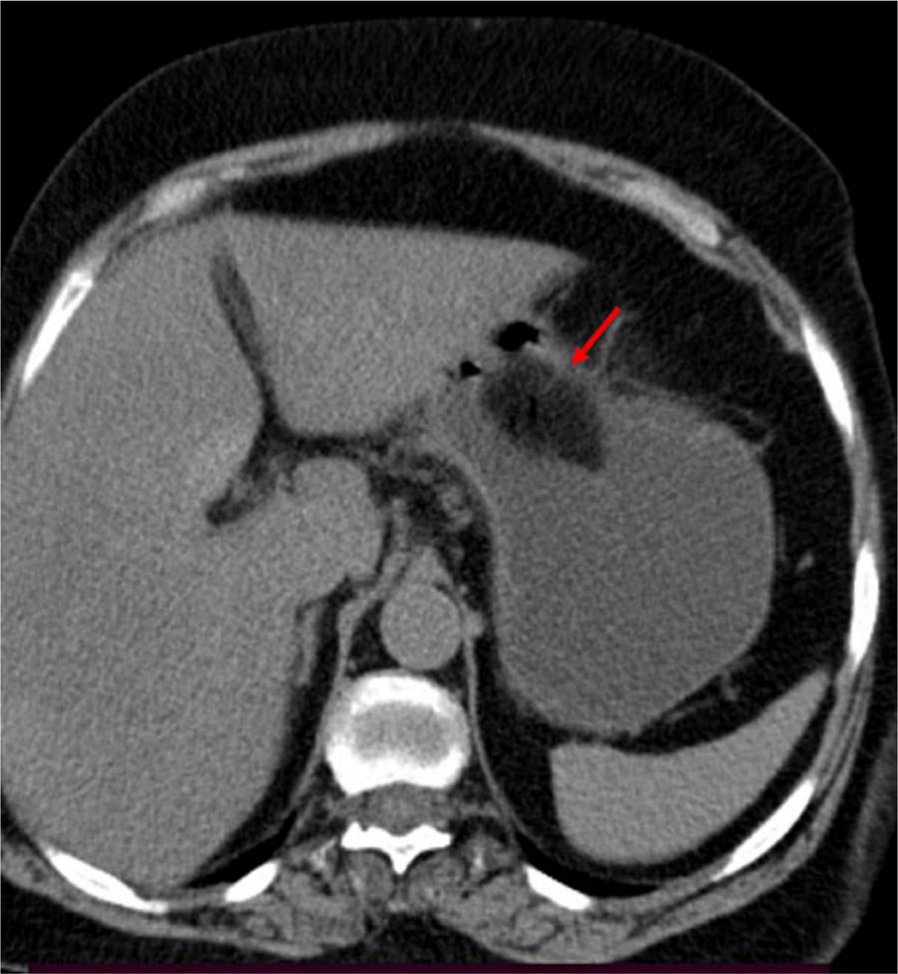
The arrow indicates a gastric-occluding intraluminal mass.

**Figure 2:**
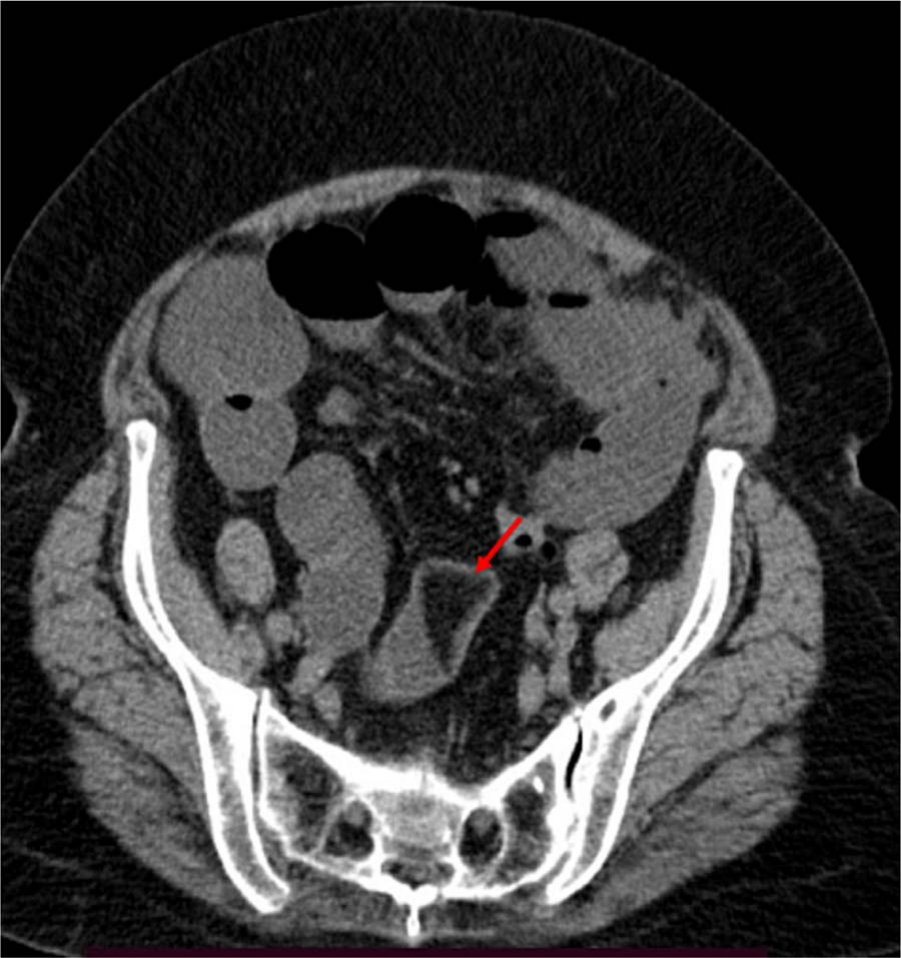
The arrow indicates an intraluminal mass in the small bowel causing proximal small intestinal obstruction.

**Figure 3:**
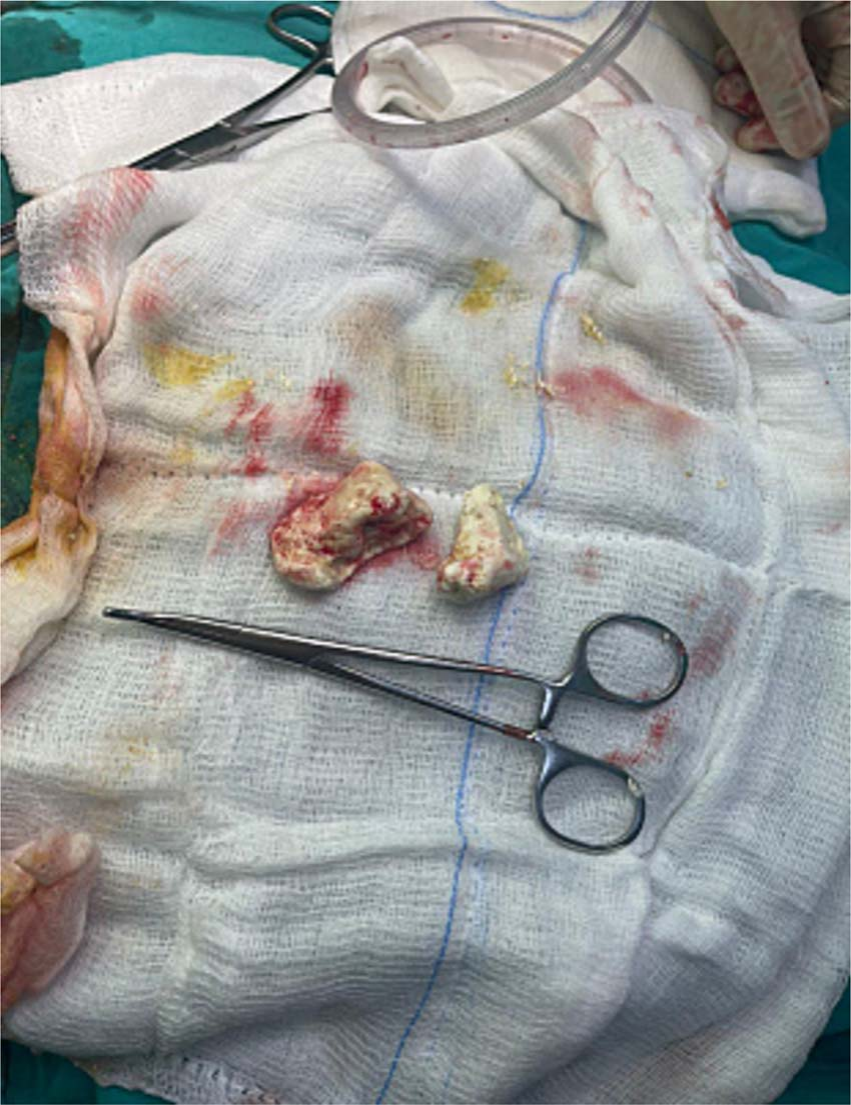
Two foreign bodies were extracted from the stomach and small bowel.

A contrast-enhanced CT of the abdomen was performed and revealed an intraluminal mass in the pylorus causing gastric dilatation (Fig. 1) and another intraluminal mass in the terminal ileum causing small bowel dilatation (Fig. 2). A nasogastric tube was inserted, and the patient was taken for exploratory laparotomy. There were significant gastric and small bowel dilatations; a gastrostomy and enterostomy were performed, and two foreign bodies were extracted (Fig. 3). The incision sites were then repaired.

Postoperatively, the patient recovered well, and enteral feeding was initiated on Day 3 with good tolerance. An erect abdominal X-ray performed on postoperative day 6 showed a normal appearance of the small bowel. The patient was discharged home with a plan for outpatient surgical follow-up.

## Discussion

According to reports, the incidence of obstruction caused by bezoars ranges between 0.4% and 4%. Bezoars commonly form in both the stomach and the small intestine. Furthermore, bowel obstruction is the most common type of bezoar complication [2, 3, 7]. Furthermore, phytobezoar, the most frequent kind of bezoar, can cause SBO at a rate of about 4% [1, 8]. Although they can be suspected in patients with co-morbidities or mental health disorders, it can be challenging to diagnose people in good health, especially if the diagnosis is made purely on the basis of a physical examination and anamnesis [9]. In our case, we found two phytobezoars that were in the patients’ stomach and small bowel ileum. Her stomach was occluded by the first and the ileum by the second, resulting in significant gastric and small bowel dilatations.

Our patients’ clinical history examination and imaging pointed to the diagnosis of small bowel obstruction irrespective of the specific cause, and bezoars were not at this time considered. According to the literature, to establish a diagnosis of obstruction owing to a bezoar, abdominal imaging, barium enema, ultrasonography, and endoscopy can be undertaken. But these approaches may be insufficient and have a number of limitations [10]. Computed tomography is particularly effective in identifying the proper treatment modality for the detection of co-existing multiple bezoars, bowel ischemia, perforation, and other probable bowel disorders in intestinal obstruction [10, 11].

In a study of 39 patients with 54 GIT phytobezoars that underwent CT scans, 59% of them had single phytobezoars, while the other 41% had multiple phytobezoars [12]. Treatment options for gastric phytobezoars include Coca-Cola, endoscopic extraction, laparotomy, and laparoscopic intervention [13, 14]. Small intestinal bezoars are managed by surgical intervention, as these patients present with small bowel obstruction and ileus [5]. The most commonly used procedure is an enterotomy to remove the bezoar [15]. In our case, a combined gastrostomy and enterostomy were done, followed by repair of the incision sites.

## Conclusion

A combined gastric outlet and small bowel obstruction secondary to bezoars is a rare condition, and preoperative diagnosis remains challenging. Dietary modification is the best way to prevent such a condition. Non-specific symptoms of small bowel obstruction with multiple sites of gastrointestinal obstruction should raise suspicion of bezoar-induced gastrointestinal obstruction, and surgical treatment should be applied in cases of small bowel obstruction.

## Data Availability

The data supporting the findings of this study are available from the corresponding author upon reasonable request.
